# Time-Lapse Electrical Resistivity Investigations for Imaging the Grouting Injection in Shallow Subsurface Cavities

**DOI:** 10.1155/2014/178203

**Published:** 2014-01-22

**Authors:** Muhammad Farooq, Samgyu Park, Jung Ho Kim, Young Soo Song, Mohammad Amjad Sabir, Muhammad Umar, Mohammad Tariq, Said Muhammad

**Affiliations:** ^1^Department of Earth Sciences, COMSATS Institute of Information Technology, Abbottabad 22060, Pakistan; ^2^Geoelectrical Imaging Lab, Korean Institute Geosciences and Mineral Resources, Daejeon 305-350, Republic of Korea; ^3^Department of Mineral Resources and Energy Engineering, Chonbuk National University, Jeonju 561-756, Republic of Korea

## Abstract

The highway of Yongweol-ri, Muan-gun, south-western part of the South Korean Peninsula, is underlain by the abandoned of subsurface cavities, which were discovered in 2005. These cavities lie at shallow depths with the range of 5**∼**15 meters below the ground surface. Numerous subsidence events have repeatedly occurred in the past few years, damaging infrastructure and highway. As a result of continuing subsidence issues, the Korean Institute of Geosciences and Mineral Resources (KIGAM) was requested by local administration to resolve the issue. The KIGAM used geophysical methods to delineate subsurface cavities and improve more refined understanding of the cavities network in the study area. Cement based grouting has been widely employed in the construction industry to reinforce subsurface ground. In this research work, time-lapse electrical resistivity surveys were accomplished to monitor the grouting injection in the subsurface cavities beneath the highway, which have provided a quasi-real-time monitoring for modifying the subsurface cavities related to ground reinforcement, which would be difficult with direct methods. The results obtained from time-lapse electrical resistivity technique have satisfactory imaged the grouting injection experiment in the subsurface cavities beneath the highway. Furthermore, the borehole camera confirmed the presence of grouting material in the subsurface cavities, and hence this procedure increases the mechanical resistance of subsurface cavities below the highway.

## 1. Introduction

**Figure 1:**
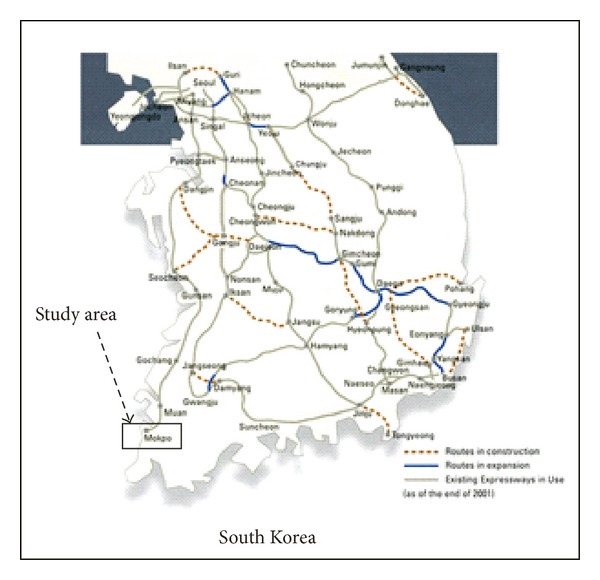
The major cities and existing expressways in South Korea are shown. The study area (Muan-gun) is pointed in the rectangular block.

A number of ground surface subsidences and sinkholes were discovered and reported in early 2005 in the residential area of Yongweol-ri, Muan-gun, south-western part of the South Korean Peninsula (Figure 1). These sinkholes caused ground surface movements and produced damage to infrastructure in the area. One of the sinkholes was approximately 10 feet wide, 8 feet long, and 4 feet deep and was discovered in the paddy field. This paddy field is just located in the vicinity of the main highway linking Yongweoli-ri and Muan-gun city. After discovery of sinkholes, detailed geological and geophysical investigations were carried out by the Korean Institute of Geosciences and Mineral Resources, Daejeon, South Korea, in 2005. The geological and geophysical investigations revealed the presence of numerous sinkholes and cavities beneath the highway [1, 2]. Hence, these investigations disclosed that geological and geophysical investigations were not conducted prior to the design or construction of the highway. The presence of sinkholes in the limestone is therefore unanticipated beneath the highway in the study area. When the stability of a highway is endangered due to sinkholes, one of the main worries for civil engineers is to determine the appropriate remedial action plan. Most of geotechnical engineers suggest cement based grouting to fill and densify the subsurface cavities. In the study area, cement based grouting was injected in order to densify or reinforce the limestone cavities beneath the highway. Geophysical techniques have produced astonishing results in case of geohazard investigations and groundwater resources in karst areas for decades. The plus point of geophysical techniques is that they are nondestructive and cost effective than direct drilling. Nevertheless, the geophysical techniques do not directly sense the desired target; they exploit the physical properties of the subsurface geological medium such as dielectric constant, density, velocity, and electrical resistivity. Electrical resistivity technique is commonly applied for subsurface sinkholes and subsidence area. Several field investigations have shown the potential use of electrical resistivity technique in detecting subsurface sinkholes and subsidence. Indeed, several researchers have successfully applied electrical resistivity imaging surveys to map karst hazards such as conduits, voids, sinkholes, and subsidence area [3–5]. Farooq et al.'s [2] investigation results pointed out the applicability and efficiency of electrical resistivity imaging to map karst structures due to the strong contrast in electrical resistivities between water filled cavities and host bedrock. Moreover, they reported that dipole-dipole array has shown the best results in identifying the subsurface cavities filled with clay/water. In a time-lapse electrical resistivity context, researchers are interested in assessing the changes in the nature of physical properties of subsurface at certain depths at different times. A major advantage of time-lapse electrical resistivity surveys is possibly improved discrimination that results from interpretation of electrical resistivity data with respect to known background state. Time-lapse electrical resistivity technique has been used by several researchers. They have reported several useful applications such as transportation process of contaminants, saline trance injection, and groundwater aquifers investigations [5–8]. In order to reinforce the sinkholes beneath the highway in the study area, cement based grout was injected in the subsurface cavities. Time-lapse electrical resistivity surveys were carried out to monitor the grouting injection and consolidation process. It is expected that time-lapse surveys effectively enable imaging of the cavities modification beneath the highway, regarding changes in moisture content. Time-lapse electrical resistivity method allows enhancing the monitoring and treatment in real time. Time-lapse electrical resistivity surveys were acquired in three stages, pregrouting, during grouting, and postgrouting injections.

## 2. Study Area

**Figure 2:**
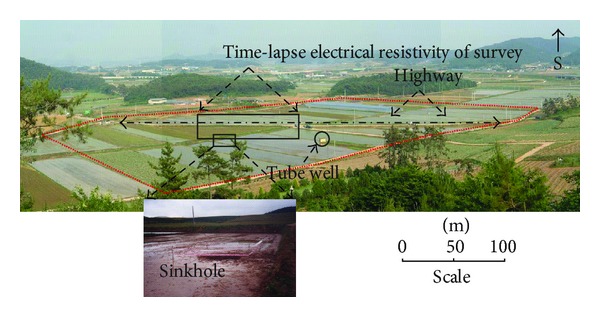
The figure depicts the location of sinkhole in the vicinity of highway and tube well for irrigation purpose at the study site in Yongweol-ri. Time-lapse electrical resistivity surveys were carried out in stages along the highway having orientation east-west.

The study area is located in Yongweol-ri, a small town in the southwestern part of South Korean Peninsula. In the study area, the ground subsidence has been reported many times in the past. The ground subsidence is one of the major problems for the citizens living in the area. Moreover, it is also a challenging task for civil engineers to resolve the problem. The subsurface cavities are present in limestone bed, which is covered by a 5–7-meter thick layer of alluvial deposits. The large Gwangju fault, geological joints, and abundant of groundwater has led to significant cavities development [2]. 100 × 50 meter area have been selected for ground reinforcement beneath the highway as shown in Figure 2. Time-lapse electrical resistivity surveys were conducted to monitor subsurface cavities conditions before, during, and after the grouting injection.

## 3. Materials and Methods

### 3.1. Ground Modification Using Grouting

When the safety and stability of infrastructure is in danger, ground reinforcement is often required. Different techniques can be used for ground reinforcement such as excavation and replacement, compaction piles, and grouting injection. The selection of appropriate ground reinforcement or modification techniques depends on several factors such as depth of water table, subsurface soil conditions, and maximum degree and depth of compaction. Dynamic compaction has provided very good results in the unsaturated zone; moreover, this technique has not provided and produced good results in case of water table at shallow depths [9]. Hence, it is important to find out alternative techniques for increasing the density of subsoil at shallow depths. Ground reinforcement with cement based grouting under high pressure has been widely used in the construction industry. Ibragimov [10] stated that cement based grouting technique is sufficiently economic and easy; moreover this technique is environmental friendly. Grouting materials by impregnation in granular media improve the mechanical properties of subsurface soils. As grouting materials enter into the granular media, they reduces pore size and alter pore structure of subsurface soil. Hence, this mechanism improves the mechanical properties of soils, such as stiffness and strength. Grouting technique has produced very excellent results in the construction and underground tunnels industry. In the study area, an attempt is made to improve the subsurface cavities beneath the highway by using cement based grouting. The grouting materials have been prepared by mixing of water, cement, and bentonite. The grouting materials were injected with high pressure into the subsurface cavities beneath the highway. In the early stage of the grouting injection process, the injection pressure tends to increase, but later, the injection pressure tends to decrease due to interconnectivity of limestone cavities. Hence, the injection material moves along the crack of the limestone cavities beneath the highway. Geotechnical engineers working on test side added sand to the mixed materials. The new mixed materials were injected into the limestone cavities until the pressure in the injection pipe was uniform.

### 3.2. Electrical Resistivity Technique

**Figure 3:**
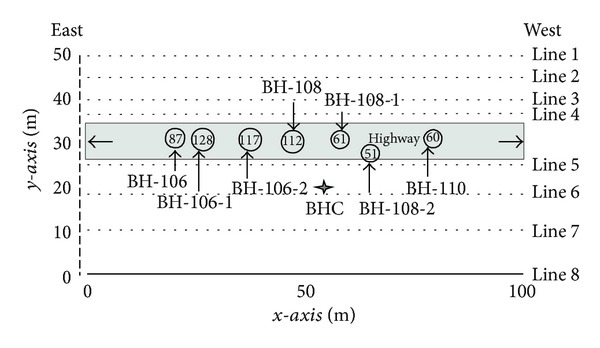
The figure depicts the orientation and length of the time-lapse 2D electrical resistivity survey. The circles indicate the boreholes location and amount of grouting in (m^3^), injected beneath the highway. The location of borehole camera (BHC) is shown by (star).

In the study area, time-lapse electrical resistivity surveys were performed where underground cavities have been reported by [2]. A total of eight survey lines have been used to cover the area of 100 × 50 meters. The survey lines were oriented east to west direction. The maximum length of survey lines is about 100 meters. The electrode spacing between the poles was 5 meters. The coordinate of each electrode and orientation of electrical resistivity lines were precisely obtained using GPS. The survey lines are shown in Figure 3. All the data was acquired using Super Sting R8/IP meter (AGI Company, USA). The maximum examined depth was approximately 25 meters. In order to obtain high quality data, dipole-dipole array was used. The software DIPROWin was developed by Korean Institute of Geosciences and Mineral Resources (KIGAM) and was used to find out the electrical resistivity distribution inversion models. The active constraint balancing (ACB) method was used to code the DIPROWin which is based on 2.5D finite element modeling and smoothness constrained least squares inversion [11]. The RMS errors of each line were carefully noted down which range from 0.02 to 0.07. Park et al. [1] reported that the general electrical resistivity values in the Yongweol-ri area range from 50 to 1000 Ohm-m. The shallow subsurface alluvial deposits are comprised of clayey soils. The limestone cavities are roofed by alluvial deposit. Moreover, the subsurface cavities are distributed in all over the area. When evaluating the safety beneath the highway, it is important to determine the exact depth and orientation of subsurface cavities underneath land surface. Park et al. [1] and Farooq et al. [2] determined the electrical resistivity distribution from all the survey lines acquired in the study area. Their results suggested that regolith layers have low resistivity values as alluvial deposits are comprised of clayey soils. However, their results also stated that the low resistivity zone at a depth of 10 meters is closely related to the limestone cavities. The drilling results confirmed the interpretation of electrical resistivity surveys of [1, 2]. Both results stated that the limestone cavities are mostly filled with groundwater and clayey soils.

### 3.3. Field Procedure

In order to reinforce the subsurface cavities beneath the highway, KIGAM officials met with local representatives, and grouting injection procedures were discussed. Seven locations were selected for grouting injection to reinforce the subsurface cavities beneath the highway. At the selected locations, 3.5 in diameter pipe was placed at the depths ranging from 5 to 12 meters. The aim of this injection was to fill the subsurface cavities with grouting materials. Grouting injection and pressure were carefully monitored and recorded during the entire operation. Ground in the vicinity of injection area was carefully monitored. A total of 616 m^3^ of cement based grout was pumped during a three-weeks period.

Electrical resistivity survey was performed before grouting injection; the initial conditions of subsurface ground are clearly described by Farooq et al. [2]. The results of Farooq et al.'s [2] interpretation suggested that numerous cavities are located in the vicinity of highway. Before and during the grouting injection work, time-lapse electrical resistivity measurements were performed at regular time interval. The percentile variations in the 2D electrical resistivity distribution during the grouting injection are displayed through 2D models, comparing the results with pregrouting electrical resistivity survey. Finally, a postinjection electrical resistivity survey was carried out to evaluate the final condition of the subsurface cavities after the grouting injection for the ground reinforcement.  Table 1 illustrates the schedules of time-lapse electrical resistivity monitoring in relation to total amount of injected grouting materials in the subsurface cavities below the highway.

## 4. Results and Discussion

### 4.1. Pregrouting Injection Electrical Resistivity Measurement Results

**Figure 4:**
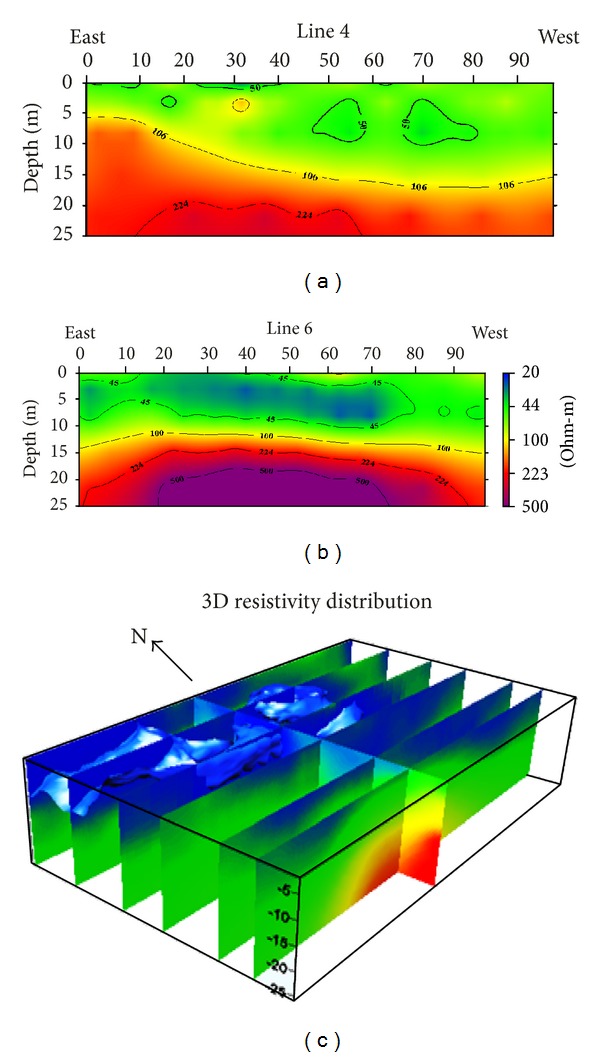
Electrical resistivity results at the pregrouting injection stage: (a) inverted 2D electrical resistivity of line 4; (b) inverted 2D electrical resistivity of line 6; (c) 3D electrical resistivity distribution at the test site; the cavities are located in the north side of the study area.

The results of pregrouting 2D electrical resistivity of especially lines 4 and 6 and 3D electrical resistivity of lines 1 to 8 show the distribution of electrical resistivity in the area as depicted in Figures 4(a), 4(b), and 4(c), respectively. Figures 4(a) and 4(b) results depict the existence of two or three main lithological layers; moreover, the lithological interface is varying from line to line.

Result of line 4 (Figure 4(a)) shows the presence of two lithological units; the upper zone having moderate resistivity values (50–70 Ohm-m) is interpreted to characterize unconsolidated material comprising of clayey sand and sand gravel admixture. The lower zone with higher electrical resistivity >100 Ohm-m is interpreted to represent the weathered limestone. Moreover, these results were confirmed by standard Penetration test (SPT) and borehole drilling in the vicinity of line 4. Line 4 electrical resistivity distribution result is coherent with SPT and borehole drilling.

Figure 4(b) exhibits the result of line 6. This line shows three distinct electrical resistivity zones. The upper zone having moderate resistivity values (40–60 Ohm-m) is interpreted to characterize unconsolidated material containing clayey soil and sand/gravel admixture. The deeper zone with high electrical resistivity values >100 Ohm-m in line 6 is interpreted to denote weathered limestone. The lowest electrical resistivity between upper and lower zone is interpreted as cavities having very low resistivity values. Furthermore, SPT and drilling were conducted on line 6 which confirmed the presence of cavities at 5–10 meters depth. In order to obtain overall subsurface condition, 3D electrical resistivity distribution of the study area is shown in Figure 4(c). The result shows the presence of numerous cavities in the north part of the study area.

### 4.2. During Grouting Injection Electrical Resistivity Measurement Results

Ground reinforcement started using grouting materials at 7 locations beneath the highway along line 4 and line 6.

To evaluate the effect that grouting injection had on the bulk electrical resistivity, post grouting injection estimated electrical resistivity is subtracted from the pregrouting injection one and normalized the result with respect to the latter according to (1). By doing so, the variation can easily be determined and interpreted in terms of orders of magnitude above or below the estimated electrical resistivity of pregrouting injection. The following equation is used to find out the variation in electrical resistivity:

(1) $$\begin{array}{c} \frac{\rho 2-\rho 1}{\rho 1}, \end{array}$$

with pregrouting electrical resistivity of subsurface being (*ρ*1) and postgrouting electrical resistivity of subsurface being (*ρ*2). Electrical resistivity on lines 4 and 6 was acquired to assess the changes produced in the subsurface cavities due to grouting injection. Here, phase 1 (Ph1) represents pregrouting injection condition, and phases 2, 3, and 4 represent during grouting or postgrouting injection condition. Equation (1) was used to calculate the variation in electrical resistivity during the grouting injection.

**Figure 5:**
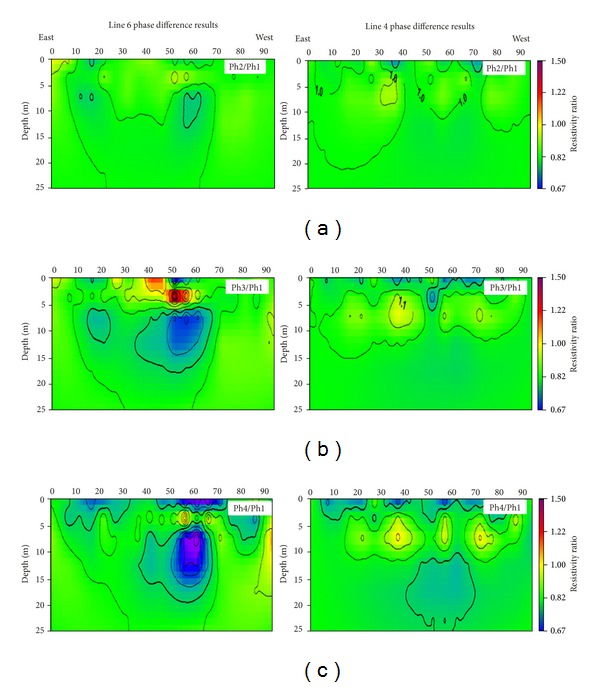
Electrical resistivity results during grouting injection stage: ((a), (b), and (c)) inverted 2D electrical resistivity of line 4 and line 6 during phases 2, 3, and 4, respectively; the results suggest that grout material moved toward the north side of the study area.

The results are shown in Figures 5(a), 5(b), and 5(c).  Figure 5(a) displays the electrical resistivity distribution along lines 4 and 6 during phase 2 (phase 2 the is starting stage of grouting injection experiment). The electrical resistivity distribution below lines 4 and 6 are shown in terms of percentile variations from the preliminary values. Positive increments (red range in the color scale, increase in electrical resistivity values) are observed in those areas of subsurface where the grouting material removed the groundwater. On the other end, negative increments (blue range in the color scale, decrease in electrical resistivity values) are evidence increments in saturation of subsurface area where water is replaced by grouting material.

Hence, these images are thus indirectly tracing the movements of grouting material in the subsurface cavities.

Table 1 illustrated the schedule of grouting injection experiment and periodical measurements of electrical resistivity survey. Figures 5(a), 5(b), and 5(c) represent the different stages of grouting injection experiment during the ground reinforcement. Figures 5(b) and 5(c) clearly show the low electrical resistivity values at the depth between 5 and 13 meters; this zone occurs between horizontal distances of 50 to 70 meters on line 6. On the other hand, Figures 5(b) and 5(c) on line 4 do not show significant changes in electrical resistivity values. As pregrouting 3D electrical resistivity distribution suggested that subsurface cavities are located in the north-western part of the study area. The interpretation results of lines 4 and 6 suggested that the grouting material moved toward the north-western side of the study area.

### 4.3. Postgrouting Electrical Resistivity Measurement Results

**Figure 6:**
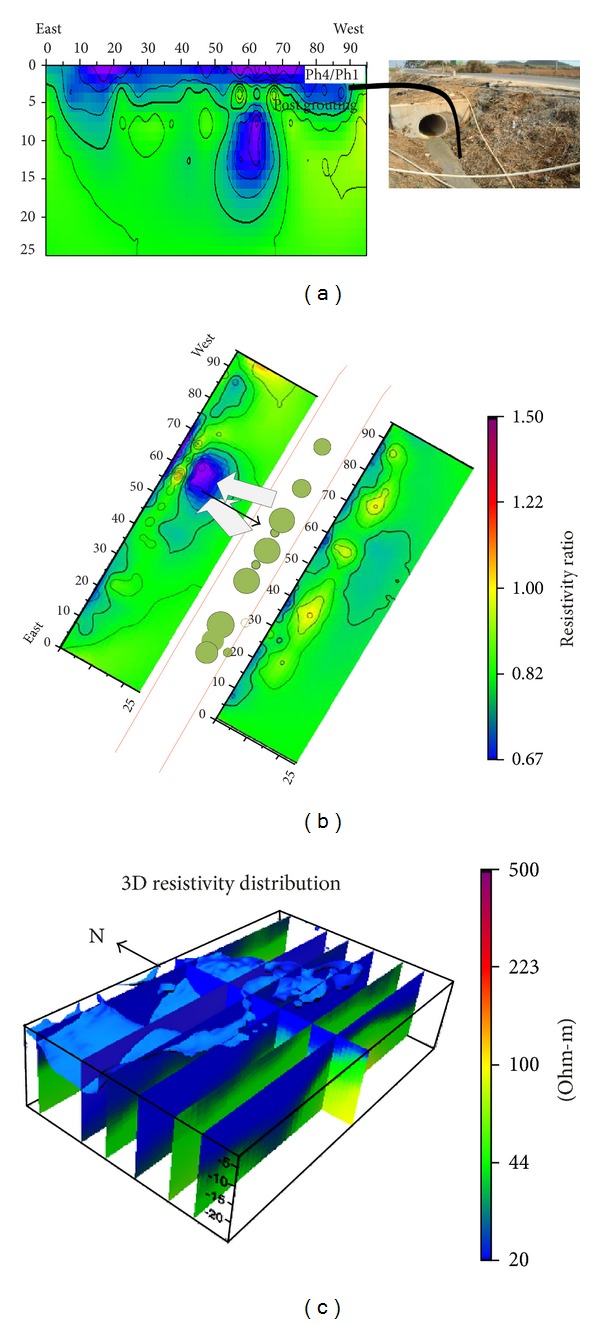
The postgrouting electrical resistivity results at test site: (a) inverted 2D electrical resistivity of line 6 when the grouting material overflows from subsurface cavities; (b) the results show movement of grout material toward line 6; (c) postgrouting 3D electrical resistivity distribution at the test site; the 3D resistivity distribution suggest that grout material moved toward the north side of the study area.

Figures 6(a), 6(b), and 6(c) display the postgrouting stage of the experiment. The results show the prominent modification steady variation in the electrical resistivity distribution. Figures 6(a) and 6(b) display the movements of grouting material toward the north as the subsurface cavities are well interconnected on that side of the study area.

Figure 6(a) clearly depicts the overflow of grout material which is also shown on the photograph.

**Figure 7:**
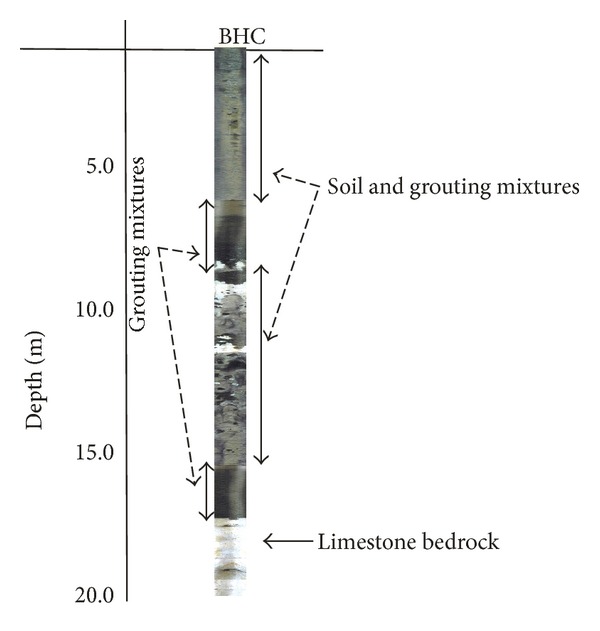
Borehole camera results; these images clearly depict the location of soil-cement mixtures, cement grout, and bedrock.

Figure 6(c) displays the result of postgrouting 3D electrical resistivity distribution, the result suggested that grouting materials spread in the north side as the subsurface cavities are well interconnect on that side of the study area.  Figure 7 shows the results of borehole camera; the results show the distribution of cement grout at different depth.

## 5. Conclusions

In this research work, we confirmed the practicality of electrical resistivity technique in the identification of subsurface cavities beneath the highway. Moreover, the time-lapse electrical resistivity surveys have depicted its ability to monitor the grouting injection beneath the cavities for the reinforcement of the subsurface cavities beneath the highway. The obtained results of time-lapse electrical surveys have shown its efficiency to map the subsurface cavities filled with grouting material, additionally, the electrical resistivity is cost effective technique.

The grouting material is expected to be linked with improvement of mechanical properties of subsurface cavities. The conclusions of this research work confirmed effectiveness of electrical resistivity for subsurface cavities characterization and monitoring of grouting injection material. However, it is recommended to carry out controlled experiment in order to understand the grouting injection experiment properly.

## Figures and Tables

**Table 1:** Time-lapse electrical resistivity monitoring and amount of grouting material.

Phase	Electrical resistivity measurement	Injection condition	Amount of grouting material (m^3^)
Phase 1 (pregrouting)	Lines 1–8	Pregrouting	0
Phase 2 (during grouting)	Lines 4 and 6	During grouting	105
Phase 3 (during grouting)	Lines 4 and 6	During grouting	325
Phase 4 (during grouting)	Lines 4 and 6	During grouting	186
Phase 5 (postgrouting)	Lines 1–8	Postgrouting	

			Total = 616 m^3^
